# Association between Coffee Consumption and Polycystic Ovary Syndrome: An Exploratory Case–Control Study

**DOI:** 10.3390/nu16142238

**Published:** 2024-07-11

**Authors:** Aïcha Meliani-Rodríguez, Ana Cutillas-Tolín, Jaime Mendiola, María Luisa Sánchez-Ferrer, Ernesto De la Cruz-Sánchez, Jesús Vioque, Alberto M. Torres-Cantero

**Affiliations:** 1 Division of Preventive Medicine and Public Health, Department of Public Health Sciences, School of Medicine, University of Murcia, El Palmar, 30120 Murcia, Spain; aicha.melianir@um.es (A.M.-R.); jaime.mendiola@um.es (J.M.); amtorres@um.es (A.M.T.-C.); 2 Institute for Biomedical Research of Murcia, IMIB-Arrixaca, El Palmar, 30120 Murcia, Spain; marisasanchez@um.es (M.L.S.-F.); erneslacruz@um.es (E.D.l.C.-S.); 3 Department of Obstetrics and Gynecology, Virgen de la Arrixaca University Clinical Hospital, El Palmar, 30120 Murcia, Spain; 4 Public Health and Epidemiology Research Group, San Javier Campus, University of Murcia, 30720 Murcia, Spain; 5 Instituto de Investigación Sanitaria y Biomédica de Alicante, Universidad Miguel Hernández (ISABIAL-UMH), 03010 Alicante, Spain; vioque@umh.es; 6 Consortium for Biomedical Research in Epidemiology and Public Health (CIBER Epidemiología y Salud Pública, CIBERESP), Instituto de Salud Carlos III, 28029 Madrid, Spain; 7 Department of Preventive Medicine, University Clinical Hospital Virgen de la Arrixaca, El Palmar, 30120 Murcia, Spain

**Keywords:** polycystic ovary syndrome (PCOS), coffee, hyperandrogenism, hyperinsulinemia

## Abstract

Polycystic ovary syndrome (PCOS) is a leading cause of infertility, with an estimated worldwide prevalence between 5% and 15%. We conducted a case–control study with 121 PCOS patients and 155 controls to assess the association between coffee intake and the presence of having a diagnosis of PCOS in women in Murcia, Spain. The PCOS diagnosis was determined following Rotterdam criteria (the presence of two of the following three conditions: hyperandrogenism, oligo-anovulation, and/or polycystic ovarian morphology). Coffee consumption was assessed using a validated food frequency questionnaire. Adjusted odds ratios (ORs) and 95% confidence intervals (CIs) were estimated using multiple logistic regression. Coffee consumption was categorized into never, less than one cup per day, one cup per day, and two or more cups per day. We found a significant inverse linear trend: the higher the coffee consumption, the lower the probability of having PCOS in multivariable analysis (*p*-trend = 0.034). Women who presented with PCOS were less likely to drink one cup of coffee compared to those who had never drunk coffee (OR = 0.313, 95% CI: 0.141–0.69). The consumption of at least one cup of coffee per day may be associated with a decrease in PCOS symptoms.

## 1. Introduction

Polycystic ovary syndrome (PCOS) is one of the most common lifelong endocrine dysfunctions in women of reproductive age [1]. It is a disorder of gonadotropin hormonal synthesis that causes ovulatory problems, insulin resistance, and excessive body fat. PCOS is also a risk factor for other metabolic and psychological disorders, such as hyperinsulinemia and type 2 diabetes [2], obesity [3], cardiovascular disease [4], gut microbiota dysbiosis [5], decreased quality of life [6], and mental health problems, such as depression, anxiety, eating disorders, and postpartum depression [7]. It is estimated that the healthcare-related costs of PCOS exceed USD 15 billion yearly in the US, with direct healthcare annual costs for mental-health-related PCOS disorders of USD 4 billion in 2021 [7]. In a Bayesian modeling study, based on that 26% of the PCOS population may develop diabetes in the United Kingdom, the cost to the National Health System has been estimated to be at least GBP 237 million per year from 2014 to 2039 [8]. The prevalence of PCOS has been difficult to estimate due to differences in the diagnostic criteria used. In Spain, the reported prevalence ranges between 5% and 10% [9,10,11]; although, worldwide, up to 15% of women of reproductive age might be affected, with an increasing trend over time in Western populations [12,13].

The etiopathogenesis of PCOS is multifactorial and includes genetic, epigenetic, and environmental factors, such as diet and lifestyle [1,9]. A systematic review found that women with PCOS have a lower overall dietary quality and poorer dietary intakes, resulting in higher cholesterol and lower magnesium and zinc levels when compared to those without PCOS [14]. Indeed, lifestyle interventions through diet, exercise, and a combination of both are the first line of action in clinical practice guidelines for the improvement of signs and symptoms of PCOS [15,16], though the long-term effect of these recommendations in clinical practice is unknown. Another frequently used intervention is a low-carbohydrate diet [17,18] with less than 45% of the total daily energy intake from carbohydrates. Given the paramount importance of insulin receptors and compensatory hyperinsulinemia in the induction of androgen excess in PCOS women, low-carbohydrate diets reducing the levels of glucose, insulin, IGF-1, and insulin-like growth factor-binding protein 1 (IGFBP1) may improve hyperandrogenism-related symptoms [18,19]. Combined calorie restrictions and physical activity interventions are the most commonly used interventions to improve the symptoms of PCOS [20]. There is scant literature on whether a specific diet or particular foods are protective factors for PCOS.

Coffee is a food that could reduce PCOS symptoms through several pathways. Coffee is one of the foods with the highest polyphenol content [21,22]. Polyphenols have been shown to be effective in improving insulin sensitivity and reducing insulin hypersecretion [23,24]. As previously mentioned, insulin resistance plays a significant role in metabolic dysfunction, exacerbating symptoms [25]. Due to the complexity of the pathways involved, several molecular mechanisms determining insulin resistance in PCOS have been described [25]. One such pathway involves the regulation of phosphatidylinositol 3-kinase (PI3K) [26]. In women with PCOS, a reduced expression of the PI3K pathway has been observed to lead to decreased insulin sensitivity and impaired β-cell function [27]. This has prompted the search for interventions targeting these pathways to improve insulin signaling. Coffee consumption has been shown to influence the PI3K pathway, as it inhibits the translocation of the glucose transporter GLUT4, thereby reducing glucose uptake and, consequently, enhancing insulin sensitivity [28].

The antioxidant properties of coffee could also be used as potential therapeutics [29]. Research indicates that women with PCOS produce more reactive oxygen species (ROS) compared to those without the disease, which are associated with low-grade inflammation [30,31]. Specifically, this inflammation is mediated by TNF-α synthesis [25,32]. Several studies in vivo and in vitro have demonstrated caffeine’s ability to suppress these inflammatory responses, for example, by modulating AMPK pathways [29,33,34]. 

Furthermore, some studies suggest that coffee consumption affects the regulation of PCOS-related hormones [35,36]. In women with PCOS, there is a disruption in ovarian function, and the hypothalamic–pituitary axis is implicated due to abnormal and irregular secretion of the Follicle–Stimulating Hormone (FSH) and the Luteinizing Hormone (LH). Altered leptin levels, impacting GnRH release, further disrupt the hypothalamic–pituitary–gonadal axis and may lead to excess androgen synthesis associated with PCOS [19]. Coffee extract may reduce circulating leptin levels, ultimately contributing to enhanced steroidogenesis [37,38].

To our knowledge, the one epidemiological study that focuses on the relationship between usual coffee consumption and PCOS was published in 2021 by Wang and coworkers [22]. In this hospital-based case–control study, the investigators found that coffee consumption was a protective factor for PCOS compared to not drinking coffee. This decrease occurred in 67.8%, 73.7%, and 84.8% of patients who consumed less than 1, between 2 and 3, or more than 3 cups/week, respectively [22]. Furthermore, a small randomized clinical trial (*n* = 34) revealed a decreased testosterone level among PCOS patients compared to those without PCOS [39]. Mousavi et al. found that the consumption of 400 mg of green coffee decreased plasma-free testosterone levels but not insulin levels and BMI [39]. Our objective was to assess whether there are differences in coffee consumption between women diagnosed with PCOS and those without PCOS (the control group). We posit that exploring this would allow us to assess whether coffee consumption improves metabolic status, which, in turn, may have beneficial effects on PCOS symptoms.

## 2. Materials and Methods

### 2.1. Design and Participants

**Figure 1:**
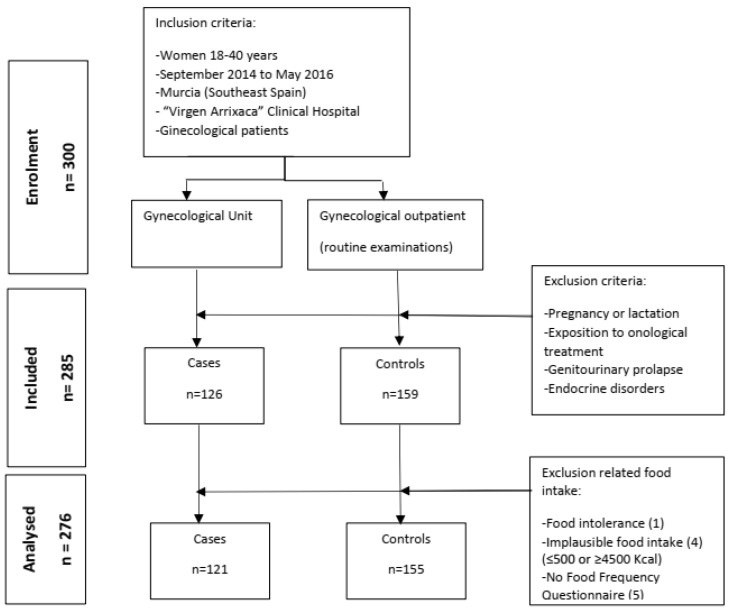
Flow diagram of the numbers of women at each stage of study.

This case–control study was conducted from September 2014 to May 2016 at the Department of Obstetrics and Gynaecology of the University Clinical Hospital “Virgen de la Arrixaca” (HCUVA), in Murcia, Spain [40]. Women were excluded if they were under 18 or over 40 years of age, pregnant or breastfeeding, undergoing cancer treatment, had genitourinary prolapse or endocrine disorders (e.g., Cushing’s syndrome, congenital adrenal hyperplasia, androgen-secreting tumors, hyperprolactinemia, and hypo- or hyperthyroidism), or had taken any hormonal medication, including contraceptives, within the 3 months preceding this study [41]. The methods have been described in greater detail in previous publications [21,42]. Figure 1 shows the number of participants enrolled, included, and analyzed. Briefly, a total of 300 gynecological outpatients were screened at the gynecology department of the HCUVA, with 285 finally included in this study [41]. The sample included 126 patients diagnosed with PCOS and 159 controls. We excluded 9 women due to incomplete Food Frequency Questionnaires (*n* = 5), those reporting an implausible energy intake (≤500 or ≥4500 kcal/day), or those who presented having a food allergy (*n* = 1). The controls were women who visited the clinic for gynecological check-ups and had no gynecological pathologies [40].

The Rotterdam criteria [43] are widely used to diagnose PCOS; we used the Rotterdam criteria in our study [1,11]. Specifically, a patient had previously been diagnosed with PCOS in the presence of two of the three following criteria [44]:

(1) Clinical and/or biochemical signs of hyperandrogenism (HA) (total testosterone level ≥ 2.6 nmol/L);
(2) Polycystic ovarian morphology (PCOM) upon ultrasound examination and exclusion of other etiologies (≥12 follicles with a diameter of 2–9 mm in both ovaries);
(3) Oligo-anovulation/amenorrhea or anovulation (OD) (menstrual cycle > 35 days or amenorrhea > 3 months).

Each patient was ascribed to one of the four clinical phenotypes of the disease [45] as follows:

- Phenotype A “HA + OD + PCOM”: hyperandrogenism + oligo-anovulation + polycystic ovarian morphology;
- Phenotype B “HA + OD”: hyperandrogenism + oligo-anovulation;
- Phenotype C “HA + PCOM”: hyperandrogenism + polycystic ovarian morphology;
- Phenotype D “OD + PCOM”: oligo-anovulation + polycystic ovarian morphology.

Phenotypes A and B are the most closely linked to metabolic disruptions, such as hyperinsulinemia, type 2 diabetes (T2DM), insulin resistance, central obesity, dyslipidemia, or metabolic syndrome [6,46]. For this study, phenotypes A, B, and D were reclassified as an “anovulatory” phenotype, phenotypes A, B, and C were classified as a “hyperandrogenic” phenotype, and phenotype C was classified as an “ovulatory” phenotype.

Identical medical exams were applied to cases and controls and included anamnesis, questionnaires, physical examination (weight, height, abdominal, and waist circumferences), and blood collection between days 2 and 5 of the menstrual cycle for hormone determination [41,43]. To assess uterine and ovarian morphology, a transvaginal ultrasound (TVUS) was performed [47]. Participants were asked to complete a series of questionnaires to identify sociodemographic characteristics, lifestyle, dietary intake, smoking and alcohol habits, family history, and psychological and social status [43].

The participants provided their informed consent for the use of their data in this study. Furthermore, the Research Ethics Committee of the University of Murcia and the HCUVA approved this study (No. 770/2013, approved 3 October 2013).

### 2.2. Assessment of Variables

For the assessment of caffeine (mg/day) and alcohol (g/day) intake, specific items of a semi-quantitative food group frequency questionnaire (FFQ) were used. We employed a validated 117-item FFQ to evaluate participants’ habitual food intake. This questionnaire has been previously validated for use in the Spanish population [48,49], and it is based on an FFQ utilized in the Nurse Health Study Cohort by Willett and colleagues [50]. This FFQ includes 101 foods divided into 7 groups: (1) dairy products; (2) eggs, meat, and fish; (3) vegetables and legumes; (4) fruit; (5) bread, cereals, and similar products; (6) oils, fats, and sweets; and (7) beverages and miscellaneous. The women were asked to indicate, for each food item, how often they had consumed the specified amount on a median basis over the past year. Specifically, regarding coffee, they were queried about how often they consumed 1 cup of coffee. The subject was asked to indicate one out of nine response options, ranging from never or once a month to more than six or more times a day. Coffee consumption was categorized into never, less than one cup per day (0.1 to 0.9 cups per day), one cup per day, and two or more cups per day. We chose this classification to enable a comparison of our results with the only previous publication conducted by Wang and colleagues [22]. Subsequently, nutrient and caffeine intake were calculated using the number of servings and grams per day multiplied by the nutritional value of each food item based on Spanish and American publications [51,52]. Nutrient intakes were adjusted for energy intake using the residual method by Willett [53].

We evaluated dietary quality by calculating the Alternate Healthy Eating Index2010 (AHEI2010) and Dietary Approaches to Stop Hypertension (DASH) as previously described [41].

To determine the level of physical activity in patients, the reduced version of the International Physical Activity Questionnaire (IPAQ-SF) was used [43]. This questionnaire quantifies the physical activity performed at different levels of intensity (vigorous, moderate, walking) and the time spent (hours/week) [54]. We also assessed tobacco smoking habits with two main questions: Have you ever smoked? If you have quit smoking, how long ago did you quit? Tobacco smoking was categorized as never smoker, ex-smoker, and current smoker.

### 2.3. Statistical Analysis

The analytic sample comprised 276 women, 121 PCOS cases, and 155 controls. The Kolmogorov–Smirnov test was used to assess the normality of variables. If variables followed a non-symmetric distribution, the Mann–Whitney or Kruskal–Wallis tests were applied, depending on whether two or more groups were compared. Data were expressed as the median and 25th and 75th percentiles for quantitative variables and as the number of subjects (*n*) and percentage (%) for qualitative variables. Differences between women with PCOS and controls were analyzed using Student’s *t*-test, the Mann–Whitney test, and the chi-square test, and the latter was used for dichotomous qualitative variables. Multivariate logistic regression models were used to test the association between coffee intake and PCOS risk. Odds ratios (ORs) and 95% confidence intervals (CIs) were calculated for each quartile, taking the first category (never) as the reference group.

Confounding was evaluated using previous knowledge of biological relevance and descriptive statistics from our study. We decided a priori that certain factors should be included based on the previous literature [41,43], such as energy intake (kcal/day), smoking (never, former, yes), and vigorous physical exercise (hours per week). Other factors were also included in multivariable models if they were associated with PCOS or/and coffee intake at a *p*-value of <0.10 or if they changed the odds ratio. When the inclusion of a potential covariate resulted in a change in the *p*-value corresponding to the coffee intake variable of less than 0.10, this covariate was kept in the final models. Therefore, the final models included age (years), energy intake (kcal/day), BMI (kg/m^2^), polyunsaturated fat (g/day), alcohol intake (g/day), smoking (never, ex-smoker, smoker), and vigorous exercise (hours/week).

Statistical analyses were carried out using IBM SPSS Statistics^®^ version 28.0.1 (IBM Corp., Armonk, NY, USA). Statistical significance was set at *p* ≤ 0.05.

## 3. Results

### 3.1. Characteristics

The mean age of the population was 29.14 (SD: 5.7) years. The mean BMI value was 24.33 (SD: 5.2), and the mean caffeine intake was 52.46 mg/day (SD: 48.5). Differences in lifestyle-related characteristics between the PCOS cases and controls have been previously published. Briefly, women with PCOS were younger (*p* = 0.001), heavier (*p* = 0.01), had a higher BMI (*p* = 0.01), and reported practicing less vigorous physical exercise than the controls (*p* < 0.01) (Table 1). However, the controls consumed more caffeine (*p* < 0.01) and alcohol (<0.01) compared to women with PCOS.

### 3.2. Characteristics by Coffee Intake

Table 2 shows the sociodemographic, physical activity, and diet-related characteristics according to coffee consumption. Women with higher coffee consumption were older (*p* < 0.001). Women who consumed one cup of coffee per day or two or more cups had a higher-quality diet, as represented by the AHEI2010 (*p* = 0.002) and DASH (*p* = 0.040). In terms of nutrient intake, we found some differences between groups. Women who never drank coffee had a lower intake of omega-3 fatty acids (g/day). Those who drank two or more cups had a higher alcohol intake (*p* < 0.001), while women who drank one cup per day of coffee had higher intakes of carbohydrates (*p* = 0.042) and sugar (*p* = 0.005) but lower intakes of total fat (*p* = 0.051).

Regarding caffeine intake by category, the median intake of women drinking one cup of coffee per day was 55.12 mg/day. The highest category, two or more cups per day, had an intake of 109.14 mg/day, while the lowest category, “never”, had an intake of 11.02 mg/day. The category of 0.1 to 0.9 cups of coffee per day had a caffeine intake of 28.49 mg/day. Other sources of coffee, such as decaffeinated coffee, tea, chocolate, and cocoa powder, were evaluated. Those who drank one cup of coffee per day also drank more chocolate (*p* = 0.011), while those in the 0.1-0.9 cups of coffee category drank more tea (*p* = 0.023) and more cocoa powder (*p* = 0.023).

The association between smoking and coffee consumption was almost significant (*p* = 0.07). We found a higher proportion of smokers among women who drank two or more cups of coffee per day (45.3%), while those who never drank coffee had a higher proportion of women who had never smoked (56%).

### 3.3. Association between Coffee Intake and PCOS

Multivariable-adjusted ORs and 95% CIs for coffee consumption adjusted for age, energy intake, BMI, polyunsaturated fat, alcohol intake, smoking, and vigorous exercise are presented in Table 3. We found a significant inverse linear trend; the higher the coffee consumption, the lower the probability of having PCOS (*p*-trend = 0.034). We can observe this association for total PCOS (*p*-trend = 0.034) and the anovulatory (*p*-trend = 0.019) phenotype. Specifically, the participants in the highest coffee consumption category (two or more cups) were 69% less likely to be a PCOS case than participants who never consumed coffee (OR = 0.31; 95% CI 0.14–0.69; *p* = 0.004). Likewise, the decrease between the highest and lowest categories was 71% and 72% for the anovulatory phenotype (OR = 0.29; 95% CI 0.12–0.72; *p* = 0.007). For the ovulatory phenotype, the relation was borderline (*p* = 0.055).

Only for the anovulatory phenotype did we find that women who drank one cup of coffee per day were 72% less likely to have PCOS compared with those who did not drink coffee (OR = 0.28; 95% CI 0.11–0.76).

## 4. Discussion

In this study, we found an inverse association between coffee consumption and the presence of PCOS. This trend held true for the subgroup with the anovulatory phenotype.

These results are consistent with the only study published to date that relates the number of cups of coffee consumed per day to the risk of PCOS. Like our study, the investigators reported an inverse linear trend, i.e., a lower risk of PCOS with increasing coffee consumption [22]. In both studies, participants who consumed approximately two cups of coffee per day were 70% less likely to have PCOS than participants who never drank coffee. 

A biologically plausible explanation for why coffee consumption could play this protective role is the possible effect on the metabolism of sex hormones in plasma. Several studies have examined the existence of a relationship between caffeine and the metabolism of certain sex hormones, such as testosterone [21,24,35,36,39,55,56]. In a randomized controlled trial, Wedick et al. found that coffee intake for four weeks could reduce total testosterone levels in overweight women compared to a control group that did not ingest coffee [56]. Another clinical trial including women with PCOS also described this inverse association [39]. In that clinical trial, patients received a treatment consisting of a green coffee capsule (400 mg) for six weeks, after which a reduction in total testosterone levels was observed [39]. However, it should be noted that the power of the results may be limited due to the small sample size of both studies (*n* = 32 and *n* = 34, respectively). Moreover, green coffee contains less than 2% caffeine but more than 50% chlorogenic acids, which makes it very different from the normally used coffee. 

**Figure 2:**
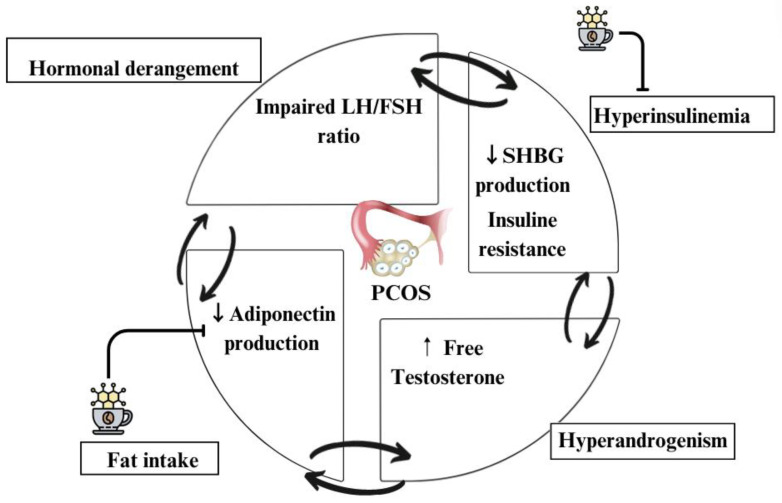
Metabolic alterations in PCOS and the influence of caffeine.

Figure 2 summarizes the metabolic changes associated with PCOS and the impact of caffeine on these changes. The double arrow symbolizes the interconnection among the impaired LH/FSH ratio, decreased SHBG, increased free testosterone, and decreased adiponectin production. Women with PCOS frequently exhibit hyperinsulinemia [15]; this may induce sex hormone-binding globulin (SHBG) inhibition. In particular, hyperinsulinemia and insulin resistance are two of the key factors for the development of PCOS [6], which impair the LH/FSH ratio. The hypersecretion of insulin has been linked to a reduction in the production of sex hormone-binding globulin (SHBG) and a consequent increase in the bioavailability of androgens in the blood, such as free testosterone [14]. This could be a risk factor for the development of PCOS, along with metabolic disturbances, such as T2DM [57]. Studies have linked caffeine intake and the protein SHBG, showing a positive association between the two [24,35,36,58]. These metabolic alterations are more commonly associated with anovulatory phenotypes [15]. Indeed, we found a statistically significant stronger association in the anovulatory phenotype. An increase in SHBG would lead to a decrease in circulating testosterone and an improvement in PCOS symptoms [59]. The results from a clinical trial in mice suggest that this could be attributed to caffeine’s ability to enhance adiponectin production, subsequently upregulating the expression of SHBG [22]. This may explain why, although caffeine consumption may increase the level of total testosterone, it is likely that most of the testosterone binds to SHBG, thus being inactivated. Similarly, some epidemiological studies also point to a protective role of caffeine in other disorders associated with PCOS, such as T2DM [24,60]. In fact, caffeine has been shown to improve insulin sensitivity as well as β-cell function in insulin secretion [58,61].

In contrast to the beneficial effect of caffeine, other studies suggest that high doses of caffeine may have the opposite effect, increasing testosterone concentration due to the inhibition of the CYP19 (aromatase) enzyme responsible for converting testosterone to estradiol [36,55]. Kousopoulos et al. observed this inhibitory activity, demonstrating reduced levels of total and free luteal estradiol in premenopausal women [55]. However, no relationship was observed in the mentioned study between coffee consumption and the concentrations of androgens or prolactin. In postmenopausal women, this effect was not observed; instead, an association with elevated SHBG levels was noted. This difference between the groups suggests that the mechanisms by which coffee regulates aromatase expression vary between premenopausal and postmenopausal women. Additionally, other factors, such as tobacco consumption, can modify coffee metabolism and its action on the aromatase enzyme. Tobacco may influence the effects of coffee by increasing its metabolism through the induction of aromatic hydrocarbons [62]. 

On the other hand, excessive caffeine consumption in coffee can have a dual effect and may potentially contribute to the development of estrogen-dependent cancers [63]. Nevertheless, coffee is associated with higher activity of two-pathway estrogen metabolites, which are less genotoxic and exhibit weaker estrogenic activity compared to the four- and sixteen-pathway estrogen metabolites, which have been linked to a higher cancer risk [63]. Furthermore, a meta-analysis on caffeine intake and breast cancer found a negative correlation between the two, which could be attributed to coffee’s potential to lower leptin levels and regulate cancer cell proliferation, and its ability to regulate proteins involved in antioxidant, detoxifying, and repair functions [64,65,66].

Although no statistically significant association was found between alcohol and the presence of PCOS in our study, it has been noted that high alcohol consumption may have reproductive consequences [67]. However, the impact of alcohol on PCOS is not entirely clear. It should be borne in mind that women with the disease are often affected by non-alcoholic fatty liver disease [68], so excessive alcohol consumption could aggravate PCOS symptoms. 

Nevertheless, we found a significantly lower presence of PCOS in those who consumed at least one cup of coffee per day, while Wang and coworkers found reduced risk across all levels of coffee intake [22]. Our subjects differed from Wang’s in that there was less heterogeneity in coffee consumption [37,69,70,71]. The disparity in effects, depending on the dose, could also be attributed to other factors, such as the variety of coffee used, the method of preparation, and even inter-ethnic variations in coffee-related metabolism [72]. CYP1A2 enzyme activity plays a crucial role in the metabolization of caffeine and is responsible for approximately 90% of this process [72]. Genetic polymorphisms that influence the rate of metabolization have been identified, with homozygous individuals showing a faster metabolization of caffeine [73]. Thus, rapid metabolizers may experience shorter caffeine effects, potentially resulting in a lesser impact on testosterone reduction. However, recent research indicates that high coffee consumption (at least three cups) is associated with the fast metabolism genotype. This association may explain the need for higher doses of caffeine in individuals with significant coffee consumption to see any effect.

However, other studies have also reported effects on hormones involved in PCOS with lower levels of caffeine [39,56]. For example, Wedick observed that drinking five cups of decaffeinated coffee resulted in a decrease in both free and total testosterone, while five cups of caffeinated coffee reduced only total testosterone. Likewise, the results obtained from decaffeinated coffee suggest that several components in coffee, other than caffeine, may be involved in this effect. Certainly, a single cup of coffee also contains numerous bioactive compounds, such as polyphenols, which are known to have advantageous effects in PCOS treatment, as they demonstrate antioxidant and anti-inflammatory properties. Polyphenols have been shown to reduce hyperglycemia, enhance acute insulin secretion and insulin sensitivity, and are utilized as a treatment to improve PCOS symptoms and effectively manage diabetes [21,74].

The current study has some limitations. Firstly, the assessment of coffee consumption was performed using an FFQ, which, like other methods employed for dietary assessment, may be subject to bias, although it should be non-differential if any exists. To improve the validity of dietary assessment, we used the validated FFQ with an average correlation coefficient for nutrient intake of 0.47 for reproducibility and 0.40 for validity. Inevitably, the information given by the patients depends on the patients’ memory, which may lead to recall bias [75]. Secondly, case–control studies have a limitation in establishing a cause-effect relationship between coffee consumption and PCOS, as they are more susceptible to selection and reporting bias. In order to reduce selection bias, cases and controls were selected from the same population. Regarding information bias, all PCOS cases were diagnosed based on the Rotterdam criteria. Thirdly, cases and controls were not matched, so we cannot rule out the possibility of residual confounding due to differences between them.

## 5. Conclusions

The results of this study suggest that the consumption of at least one cup of coffee may be inversely associated with the presence of PCOS. Coffee, abundant in bioactive compounds, has garnered interest in clinical and epidemiological research for its potential implications in anti-inflammatory effects, improved insulin sensitivity, and hormone regulation. Possible mechanisms behind this association may stem from the improvement of metabolic disorders, thus aiding in addressing the associated complications that underlie PCOS. However, additional and powered studies using causal inference methods to approximate randomization are necessary to further inform this hypothesis.

## Figures and Tables

**Table 1:** Comparison of the general characteristics of the study sample between cases with PCOS and controls (*n* = 276).

	Control (*n* = 155)	PCOS (*n* = 121)	*p*-Value
	Median (P_25_–P_75_)	Median (P_25_–P_75_)	
Age (years)	31.0 (24.25–34.75)	28.00 (23.00–32.00)	0.001
BMI (kg/m^2^)	22.2 (20.4–24.5)	24.06 (23.9)	0.01
Vigorous exercise (hours/week)	1.0 (0.0–3.8)	0.0 (0.0–1.7)	<0.001
Calorie intake (Kcal)	1770.9 (1430.2–2145.0)	1948.5 (1572.8–2304.9)	0.13
Saturated fats (g/day)	11.4 (10.0–13.9)	12.6 (11.0–14.4)	0.07
Alcohol intake (g/day)	2.3 (0.9–6.0)	1.3 (0.0–3.8)	<0.001
Caffeine intake (mg/day)	49.3 (21.5–92.1)	25.2 (11.27–53.08)	<0.001
Sex hormone-binding globulin (SHBG) (nmol/L)	44.0 (28.0–58.0)	29.0 (17.9–45.5)	<0.001
Smoking, *n* (%)	80.0 (51.6%)	60.0 (49.6%)	0.71

**Table 2:** Demographic characteristics and nutrient intake according to coffee consumption (*n* = 276).

	Never	<1 Cup/Day	1 Cup/Day	≥2 Cups/Day	*p*-Value
	*n* = 86	*n* = 70	*n* = 56	*n* = 62	
	Median (P_25_–P_75_)	Median (P_25_–P_75_)	Median (P_25_–P_75_)	Median (P_25_–P_75_)	
Age (years)	29.50 (23.75–34.00)	25.00 (22.00–31.00)	30.00 (27.00–33.00)	32.00 (27.00–35.00)	<0.001
BMI (kg/m^2^)	22.59 (20.06–27.68)	23.24 (20.84–28.92)	22.48 (20.30–26.30)	22.43 (20.99–25.64)	0.811
Moderate exercise (hours/week)	4.42 (0.25–8.50)	4.25 (0.63–10.00)	4.50 (1.50–14.00)	4.00 (1.88–7.13)	0.595
Vigorous exercise (hours/week)	0.00 (0.00–2.00)	0.00 (0.00–2.25)	0.75 (0.00–3.83)	1.00 (0.00–4.50)	0.055
Energy intake (kcals/day)	1746.01 (1398.73–2204.89)	1843.43 (1387.39–2320.01)	1865.17 (1415.97–2218.14)	1908.00 (1534.97–2572.64)	0.397
Diet quality, AHEI 2010	59.00 (53.00–59.00)	61.00 (54.40–70.25)	66.50 (55.25–72.75)	66.50 (61.25–75.00)	0.002
Diet quality, DASH	22.00 (18.00–26.00)	22.00 (19.00–25.00)	23.00 (20.00–26.75)	24.00 (20.00–29.00)	0.040
Protein (g/day)	90.21 (79.63–101.66)	88.13 (79.94–101.00)	85.48 (80.17–103.74)	94.10 (82.30–105.18)	0.243
Carbohydrate (g/day)	174.14 (143.86–192.35)	171.55 (152.08–194.09)	188.11 (165.77–210.27)	174.66 (152.69–197.81)	0.042
Total fat (g/day)	73.53 (65.62–80.89)	72.87 (63.64–77.84)	67.35 (858.32–75.78)	70.62 (64.26–78.69)	0.051
Saturated fat (g/day)	20.61 (18.44–24.56)	21.23 (16.84–24.49)	19.94 (16.23–22.82)	19.99 (16.97–22.30)	0.180
Monosaturated fat (g/day)	32.99 (29.14–38.26)	31.30 (27.92–38.32)	30.8 4 (25.69–34.12)	32.03 (28.61–37.11)	0.082
Polyunsaturated fat (g/day)	11.73 (10.25–13.78)	11.54 (10.36–14.21)	11.48 (9.86–14.41)	12.93 (10.70–14.96)	0.234
Omega-3 fatty acids (g/day)	1.38 (1.22–1.63)	1.47 (1.18–1.78)	1.40 (1.16–1.73)	1.56 (1.32–1.93)	0.048
Cholesterol (mg/day)	277.81 (230.89–346.23)	279.48 (223.01–363.74)	257.68 (207.29–308.47)	273.49 (207.35–340.55)	0.421
Fiber (g/day)	19.20 (14.95–23.62)	18.27 (15.11–23.86)	20.04 (16.34–25.95)	20.93 (17.39–25.38)	0.128
Sugar (g/day)	68.57 (58.44–83.44)	76.64 (59.93–87.50)	83.35 (65.55–102.62)	71.09 (60.12–84.19)	0.005
Alcohol (g/day)	1.20 (0.00–3.41)	2.33 (0.56–4.56)	1.70 (0.52–5.04)	3.62 (1.03–6.76)	0.009
Caffeine (mg/day)	11.02 (5.75–19.80)	28.49 (17.03–43.02)	55.12 (46.18–60.70)	109.14 (84.51–128.06)	<0.001
Other sources of caffeine					
Tea (servings/day)	0.00 (0.03–0.43)	0.00 (0.07–0.21)	0.00 (0.03–0.29)	0.00 (0.03–0.07)	0.023
Decaffeinated coffee (servings/day)	0.00 (0.00–0.07)	0.00 (0.14–0.52)	0.00 (0.00–0.12)	0.00 (0.00–0.07)	0.195
Chocolate (2 chocolates) (servings/day)	0.07 (0.00–0.14)	0.07 (0.00–0.14)	0.07 (0.14–0.43)	0.07 (0.07–0.36)	0.011
Cocoa powder	0.00 (0.00–0.21)	0.07 (0.00–0.21)	0.00 (0.00–0.12)	0.00 (0.00–0.07)	0.023
Smoking, *n* (%)					
Yes	19 (22.6)	19 (27.5)	17(32.1)	29 (45.3)	0.066
Former	18 (21.4)	36 (52.2)	10 (18.9)	15 (23.4)	
Never	47 (56)	14 (20.3)	26 (49.1)	20 (31.3)	

**Table 3:** Association for coffee consumption and polycystic ovary syndrome (*n* = 276).

Coffee, Cups	0 Cups/Day	<1 Cup/Day	1 Cup/Day	≥2 Cups/Day	*p*-Value for Trend
Median, P_25_–P_75_	0 (0–0)	0.3 (0.07–0.4)	1 (1–1)	2.5 (2.5–2.5)	
N (%)	86 (31.2)	70 (25.4)	56 (20.3)	64 (23.2)	
Total PCOS (*n* = 155)					
N cases/controls	37/49	35/35	20/36	17/47	
Crude OR	1	0.76 (0.40–1.42)	0.42 (0.21–0.84)	0.27 (0.14–0.55)	0.001
	*p*-value	0.385	0.014	<0.001	
Adjusted models ^1^	1	0.53 (0.26–1.11)	0.50 (0.23–1.09)	0.31 (0.14–0.69)	0.034
	*p*-value	0.093	0.083	0.004	
Anovulatory phenotype (*n* = 88)					
N cases/controls	37/37	28/35	10/36	13/47	
Crude OR	1	0.80 (0.41–1.57)	0.28 (0.12–0.64)	0.28 (0.13–0.59)	<0.001
	*p*-value	0.517	0.003	<0.001	
Adjusted models ^1^	1	0.55 (0.25–1.22)	0.28 (0.11–0.76)	0.29 (0.12–0.72)	0.019
	*p*-value	0.141	0.012	0.007	
Ovulatory phenotype (*n* = 33)					
N cases/controls	12/37	7/35	10/36	4/47	
Crude OR	1	0.62 (0.22–1.75)	0.86 (0.33–2.23)	0.26 (0.08–0.88)	0.164
	*p*-value	0.362	0.751	0.030	
Adjusted models ^1^	1	0.39 (0.13–1.23)	0.91 (0.32–2.57)	0.28 (0.08–1.03)	0.126
	*p*-value	0.109	0.854	0.055	

^1^ Model adjusted by age (years), energy intake (kcal/day), BMI (kg/m^2^), polyunsaturated fat (g/day), alcohol intake (g/day), smoking (never, ex-smoker, smoker), and vigorous exercise (hours/week).

## Data Availability

The data that support the findings of this study are restricted for research use only. The data are not publicly available. Data are available from the authors upon reasonable request and with permission from the Departments of Preventive Medicine and Obstetrics and Gynecology, University Clinical Hospital Virgen de la Arrixaca, Spain.

## Abbreviations

PCOS	Polycystic Ovary Syndrome
BMI	Body Mass Index
H	Hyperandrogenism
O	Oligo/amenorrhea
POM	Polycystic ovary morphology
H + O	“Hyperandorgenism + Oligo/amenorrhea” phenotype
H + O + POM	“Hyperandorgenism + Oligo/amenorrhea + Polycystic ovaries morphology” phenotype
H + O	“Hyperandorgenism + Oligo/amenorrhea” phenotype
O + POM	“Oligo/amenorrhea + Polycystic ovaries morphology” phenotype
